# Anthropometrics, physical fitness, and sport-specific performance of young German canoe sprint athletes (U13-U17) to predict senior performance level: a machine-learning approach

**DOI:** 10.1038/s41598-026-58055-3

**Published:** 2026-06-21

**Authors:** Christian Saal, Jan Willem Teunissen, Urs Granacher, Norman Helm, Torsten Warnke, Olaf Prieske

**Affiliations:** 1 Department of Health and Physical Activity, Faculty of Humanities, Otto-von-Guericke-University Magdeburg, Magdeburg, Germany; 2 Institute for Studies in Sports and Exercise, HAN University of Applied Sciences, Nijmegen, Netherlands; 3 Department of Movement and Sports Sciences, Faculty of Medicine and Health Sciences, University of Ghent, Ghent, Belgium; 4 Exercise and Human Movement Science, Department of Sport and Sport Science, University of Freiburg, Freiburg, Germany; 5 Olympic Testing and Training Centre Brandenburg, Potsdam, Germany; 6 Institute for Applied Training Science, Leipzig, Germany; 7 Division of Exercise and Movement, University of Applied Sciences for Sports and Management Potsdam, Potsdam, Germany

**Keywords:** Talent promotion, Long-term athlete development, Elite sports, Paddling, Athletic performance, Sporting success, Anatomy, Health care, Physiology

## Abstract

The aim of this study was to evaluate whether machine learning models comprising anthropometric, physical fitness, and sport-specific performance data from young canoe sprint athletes can predict their senior performance level (SPL). Between 1992 and 2019, anthropometric (e.g. body mass/height), physical fitness (e.g. 800 m/1500 m run, 2 min bench press/pull), and sport-specific performance (e.g. 250 m/2000 m on-water canoe sprint) data as well as age (i.e. U13 to U16) and sport discipline were annually examined in young male and female canoe sprint athletes (n = 729, male: 495, female: 234). A benchmark experiment was conducted to evaluate and compare multiple classification models and to use the final model to predict SPL (national vs. international) on three validation datasets (n = 103, U13 to U17, 2021 to 2023) with ground-truth labels from 2025. Findings revealed that an XGBoost model achieved acceptable discrimination (AUC = 0.81) and balanced accuracy (BACC = 0.73) for predicting SPL in young canoe sprint athletes. However, precision for the international class was low (PRAUC = 0.35, PPV = 0.20), indicating many false-positive international predictions. The most important feature was 2000 m on-water canoe sprint test. Furthermore, predictions on the three external validation datasets showed limited temporal generalizability, with moderate discrimination (AUC: 0.68 to 0.73), modest but consistently above-chance balanced accuracy (BACC: 0.59 to 0.63), and moderate but variable sensitivity (0.20 to 0.67). However, precision for identifying international athletes was low across the external validation datasets, indicating a high false-positive rate. Therefore, the model should be interpreted as an acceptable screening tool. However, low precision and variable sensitivity limit its practical utility as a stand alone selection instrument. The present findings may help practitioners involved in talent selection and development in Olympic canoe sprinting and may inform the development of future prediction models for young canoeists based on anthropometric, physical fitness, and sport-specific performance data.

## Introduction

Long-term athlete development (LTAD) starts with talent identification (TI) and continues with the development of young promising athletes through the different LTAD stages^1^. The goal is to increase the likelihood of developing young and promising athletes into senior high performance athletes^2,3^. Forecasting a young athlete’s potential at the senior level is key in TI and a prerequisite to enter talent promotion programs^3^. A talented young athlete may be defined as an individual who demonstrates exceptional potential for future elite performance within a dynamic talent development process. In line with statistical definitions of talent, such individuals typically exhibit performance capacities that are approximately two to three standard deviations above the population mean. This potential is reflected in a multidimensional profile of performance-related characteristics, rather than in current performance outcomes alone^4^. This is also a challenge, to identify young athletes who have the potential to excel at senior level using performance and anthropometrics data^5–7^. In the field of competitive and elite sports, TI criteria are often based on coaches’ subjective ratings, anthropometric, physical, psychological, sociodemographic, physiological characteristics, and competition results^8–10^. This multidimensional nature should be always considered in talent identification, and dynamic models should be used where possible when assessing the potential of young athletes^4^. Knowledge of the predictive value of such rather easy to monitor characteristics can be considered of particular importance for coaches, athletes, and researchers^11^.

In Olympic canoe sprinting, coaches consider specific anthropometric variables (e.g. body mass, body height) and physical fitness qualities (e.g. cardiorespiratory endurance, muscular endurance, pulling power, dynamic balance) to be key performance criteria for sporting success^12,13^. Additionally, psychological characteristics such as self-confidence and motivation showed high associations with canoe sprint performance and were thus included in sport-specific TI^12^. Previously, multiple linear regression models were used to predict race times in young and adult canoe sprint athletes around the time of testing period based on anthropometric and physical fitness data^6,14,15^. For instance, a large percentage of 500 m to 10,000 m race time variance (i.e. 83 to 92%) could be explained by sets of anthropometric (e.g. body mass, sitting height) and physical fitness data (e.g. cardiorespiratory endurance, muscle strength, muscular endurance)^14^. Additionally, anthropometric characteristics, physical fitness data, and sport-specific performance (i.e. race times) were found to be significantly different between canoeists selected for the state team compared with non-selected athletes in favor of the state team members^14^. A more recent study substantiated the predictive validity of anthropometric and physical fitness data on race time during competition in young national-level (tier 2 training and performance caliber^16^) canoe sprint athletes^6^. More specifically, the applied linear regression model with the highest explained variance for 500 m (r^2^ = 0.88) and 2000 m race time (r^2^ = 0.69) included the sport discipline (i.e. canoe), anthropometrics (i.e. skeletal muscle mass), and physical fitness variables (i.e. upper-limb muscular endurance, lower-limb muscle power)^6^. Similarly, it was reported that chronological age, anthropometrics (e.g. body mass, maturity status), and physical fitness (i.e. overhead medicine ball throw, sit-and-reach) significantly explained variances (r^2^) in actual race time across 200 m to 1,000 m (0.45 to 0.67) in young male national-level (tier 2) canoe sprint athletes with the factors maturity status and chronological age as best predictors^15^.

While there is evidence on race-time prediction around the testing period using anthropometric and physical-fitness data, less is known about the long-term prediction of future senior canoe sprint performance using a multidimensional set of potential predictors, including anthropometric, physical as well as psychological, and sociodemographic variables^17,18^. In fact, research on the long-term prediction in other individual sport disciplines revealed inconsistent findings^5,18–20^. For instance, a retrospective cohort study analyzed career trajectories of Italian high and long jumpers and reported that 10 to 25% of top-level adult athletes were top-level at the age of 16 (except female high-jumper), suggesting that the performance level before the age of 16 years is not a good predictor of senior performance level (SPL) in jumpers^5^. Performance data of junior and senior international swimmers competing between the 2010 Youth Olympic Games (YOG) and the Paris 2024 Olympic Games show that among all YOG swimmers, 35.1% later competed at the senior Olympic Games, while 58.7% of YOG medalists and 70.5% of YOD champions participated in the senior Olympics^18^. A recent systematic review and meta-analysis of retrospective and prospective studies confirmed that successful junior and successful senior athlete cohorts were largely independent of each other, with only about 12 to 13% of athletes being successful both at youth and at later age^21^. This finding is supported by earlier work showing that only 6 to 25% of international junior athletes reached international senior level and 10 to 32% of international senior athletes had previously competed internationally as juniors^19^. In this regard, longitudinal (i.e. repeated measurements across time) and multidisciplinary study designs (including for instance anthropometric, physical fitness, sport-specific performance data) using advanced statistical modelling (e.g. machine learning approaches) were recommended^18,22–24^ and needed. For example, a large longitudinal dataset from competitive swimming in the United States was used to predict swimming time at the age of 18 years based on swimming times at ages 10 to 15 years and the proposed Wisdom of Crowds Classifier (WoCC) achieved moderate to good precision (0.49 to 0.75) across freestyle and breaststroke prediction tasks^20^.

To the best of our knowledge, there is no previous study in canoe sprinting that has applied advanced statistical or machine-learning models using anthropometric, physical-fitness, and sport-specific performance data from young athletes to predict SPL. Therefore, it remains unclear whether machine learning models can predict senior performance level from junior data with acceptable accuracy, identify the most predictive variables of long-term success, and generalize to new athlete cohorts. Thus, the aim of this exploratory study was to evaluate whether machine learning models comprising anthropometric, physical fitness, and sport-specific performance data from young canoe sprint athletes can predict their senior performance level (SPL).

## Methods

### Study design

**Fig. 1:**
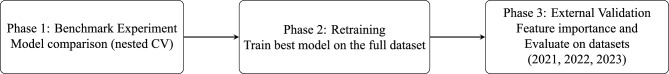
Workflow of the modelling approach. Phase 1: Benchmark experiment to compare candidate models and identify the best classifier. Phase 2: Retraining of the selected model on the full benchmark dataset. Phase 3: Feature importance and external validation on independent cohorts (2021 to 2023) to evaluate model generalization.

A retrospective cohort study of young German canoe sprint athletes was conducted using existing testing data and retrospectively ascertained SPL outcomes to develop and evaluate prediction models. As part of a national TI program of the Federal State of Brandenburg, Germany, data were collected between 1992 and 2019 (training/test set) and between 2021 and 2023 (external validation sets), comprising anthropometric, physical fitness, and sport-specific performance data. Standardized measurements of anthropometry, physical fitness, and sport-specific performance were taken annually during the preparation period (i.e. end of September/beginning of October) at the Olympic Testing and Training Center Brandenburg (Germany) in athletes nominated by their coaches as “promising”. Local weather conditions during the preparation period are typically mild, with maximum daytime temperatures of about 15 to 19°C, moderate winds of around 4 m/s , and monthly precipitation of roughly 40 to 50 mm^25^. These measurements were comparable to procedures used in German canoe sprint development programs. For each observation in the train/test, and validation datasets, the peak SPL attained by each athlete during their career was recorded and used as dependent variable. The athletes’ SPL was defined as the highest German cadre status they achieved throughout their sports careers. More specifically, athletes were classified as national (n = 670) or international (n = 59) (Table 1). In accordance with the training and performance caliber classification^16^, international athletes achieved international competition level (i.e. tier 4 and 5) including Olympic Games (n = 9), while national athletes competed at the national level or below (i.e. tier 2 and 3). First, a benchmark experiment was conducted to identify the most suitable classification model for predicting SPL, in which six models were compared. Second, the selected model was retrained on the full dataset (1992 to 2019) to obtain the final predictive model. Third, feature importance was quantified using Shapley values on the external validation sets. Finally, the model’s generalization performance was assessed on three external validation sets (Fig. 1). This study and all testing procedures adhered to the principles of the Declaration of Helsinki. The study protocol and all testing procedures were approved by the Institutional Committee of the Federal Canoeing Association of Brandenburg, Germany. Written informed consent was obtained from all subjects and/or their parents or legal guardians.

### Data

Four datasets were used. The dataset (train/test) used for the benchmark experiment and model training comprising male and female canoe sprint athletes aged 12 to 16 years (mean: ${13 \pm 0.77}$) who participated in the TI program. For the analyses, only the first available test data of each athlete was included. Resulting in a $729\times 13$ data frame (e.g. age, sport discipline [i.e. kayak female, kayak male, and canoe male], SPL, anthropometric, physical fitness, and sport-specific tests), with complete cases and each entry corresponding to an individual athlete measured once. The ground-truth labels for the train/test dataset were collected and annotated in 2020 as part of a master’s thesis. The three external validation datasets were obtained from athletes aged 13 to 17 years, assessed in 2021, 2022, and 2023 (n = 21, n = 44, n = 38, respectively) by the Federal Canoe Association of Brandenburg. Across these datasets, 27 athletes were measured once, 20 twice, and 12 three times. At the last measurement, the athletes had a mean age of ${14.7 \pm 0.99}$. Ground-truth labels are obtained in 2025 and provided by the Olympic Testing Centre Brandenburg. Athletes aged 13, in the 2023 validation dataset, were excluded because their ground truth values were yet missing. Tables 1 and 2 show the number of athletes of the train/test and validation datasets across age cohort, sport discipline, and SPL.

**Table 1:** Number of athletes from the train/test set across age cohort, sport discipline, and peak senior performance level.

Sport discipline	Senior performance level	Age				Sum
		U13	U14	U15	U16	
Canoe male	International	15	5	2	1	23
	National	125	39	13	5	182
Kayak male	International	17	3	2	0	22
	National	180	60	19	9	268
Kayak female	International	9	1	3	1	14
	National	157	37	18	8	220
Sum	International	41	9	7	2	59
	National	462	136	50	22	670
	All	503	145	57	24	729

**Table 2:** Number of athletes from the external validation sets across age cohort, sport discipline, and peak senior performance level.

Sport discipline	Senior performance level	Age					Sum
		U13	U14	U15	U16	U17	
Canoe male	International	1	2	2	1	0	6
	National	0	3	2	2	0	7
Kayak male	International	3	7	3	1	0	14
	National	14	17	12	6	0	49
Kayak female	International	0	1	1	1	0	3
	National	3	14	3	3	1	24
Sum	International	4	10	6	3	0	23
	National	17	34	17	11	1	80
	All	21	44	23	14	1	103

### Anthropometrics

Anthropometric assessment included measurements of body height, body mass, and arm span. Additionally, sitting height was determined (i.e. height from the bottom of the seat to the vertex of the head) for young male/female kayakers and in a kneeling position (i.e. height from the bottom of the ground to the vertex of the head during kneeling position) for young male canoeists^11^. Arm span width was measured with the athlete standing upright against a wall, arms extended horizontally, from the wall-aligned middle finger to the outermost point of the contralateral middle finger using a fixed tape measure^26^. Sitting and standing height are often used measures to estimate the maturity offset^27^. In the present study, sitting/kneeling height was entered as a raw anthropometric predictor. Maturity offset was not calculated and was therefore not included as a separate predictor variable.

### Physical fitness

#### Muscle power

The underhand ball throw test was used to determine proxies of muscle power in young canoe sprint athletes. Previously, high test-retest reliability was reported with an intraclass correlation coefficient (ICC) of 0.95^28^. For testing, athletes used a steel/rubber ball with 3 kg for girls and 5 kg for boys. The participating athletes were in upright standing position and held the ball with both hands in front of the body. Starting from the standing position, the athletes had to perform an underhand countermovement swing which was immediately followed by throwing the ball as far as possible in horizontal direction. After the ball push-off, the feet had to remain on the ground. Trials with jumps were discarded. Three trials were performed, and the best trial in terms of maximal horizontal throwing distance was used for analysis.

#### Muscular endurance

Muscular endurance was determined by means of the 2 min bench press and bench pull tests. Test-retest reliability was high for the bench press (ICC = 0.94) and the bench pull (ICC = 0.96) tests in young canoe sprint athletes^6^. When in supine or prone position on a bench, participants had to push or pull a loaded barbell for 2 min with as many repetitions as possible. Athletes and their coaches individually selected the barbell load based on previous test performances in an attempt to maximize mechanical work. Depending on age group and sex, these maximum loads ranged from 17.5 to 35.0 kg in female athletes and from 17.5 to 47.5 kg in male athletes. A digital linear encoder was attached to the barbell to register the total distance covered. Only correctly executed repetitions that reached the defined turning points were counted. Repetitions with hip lift, excessive lumbar extension, or without contact at the turning points were excluded. Finally, a strength index was calculated for both exercises as load (kg)$\times$ repetitions $\times$ displacement (cm), with displacement measured as the vertical distance from the top of the resting barbell to the underside of the board.

#### Cardiorespiratory endurance

Cardiorespiratory endurance was assessed using an on-land running test on a regular 400 m outdoor track over 800 m for female athletes and 1,500 m for male athletes using a hand-held stopwatch. The sex-specific running distances were predefined by the standardized testing system used in the canoe sprint talent development program. No pacing strategy was prescribed, and athletes completed the test using self-selected pacing. High test-retest reliability was reported for this test protocol^6^. The athletes in the respective age groups started from a standing position after an acoustic signal was provided by the tester. One trial was allowed and used for analysis.

#### Linear sprint speed

A 30 m on-land sprint test was used to determine linear sprint speed using photoelectric timing gates (Tag Heuer, model CP505, La Chaux-de-Fonds, Switzerland). High test-retest reliability was reported (ICC = 0.94)^6^. The start was initiated individually without an external start signal, with a run-up of 5 m (i.e. flying start). The photoelectric timing gates were positioned at approximately knee height and aligned on a straight line at the start and end of the 30 m timed section. The best out of three trials in terms of the fastest running time was included in the analysis.

### Sport-specific performance

Sport-specific performance was quantified using 250 m and 2,000 m on-water canoe sprint tests. Race time in the respective sport discipline boat (i.e. kayak, canoe) was recorded with a stop-watch. The participants had to complete the 250 m trial first (i.e. pairwise start) followed by a 30 min break and the 2,000 m trial (i.e. single start). The 2,000 m time trial was completed on a 1,000 m track with a u-turn to account for possible effects of weather conditions (e.g. wind).

### Data processing and statistical analyses

Continuous predictor variables comprising anthropometric, physical fitness, and sport-specific performance data. Fitness and sport-specific performance scores were transformed using an interval-scaled scoring system to provide identical measurement units. Additionally, two categorical predictor variables (i.e. sport discipline [i.e. combined sex and boat type], SPL) were applied. The interval-scaled scores were derived using a point-based scoring system that is proprietary to the Federal Canoe Association of Brandenburg and is not publicly available. The proprietary transformation was monotonic and stepwise linear within tests and applied using age-, sex-, and discipline-specific normative tables. Normative data have been reported elsewhere^11^.

#### Benchmark experiment

We conducted a benchmark experiment using the train/test dataset and the R package MLR3^29^ to evaluate and compare multiple classification models. To account for the class imbalance, stratified cross-validation was applied by defining the target variable as stratum, ensuring that the proportion of international and national athletes was preserved in each resampling fold (overall class distribution: 8% international vs. 92% national). The models included in the analysis were: (1) Recursive Partitioning (Rpart), (2) Random Forest (Ranger), (3) Featureless Classification, (4) Random Forest tuned (Ranger tuned on area under curve (AUC)), (6) Random Forest tuned (Ranger tuned on balanced accuracy (BACC)), (5) XGBoost (XGBoost tuned on AUC), and (7) XGBoost (XGBoost tuned on BACC). The non-tuned models were evaluated with default settings. The tuned models were constructed using a graph of multiple pipeline operations, incorporating class balancing and a data processing pipeline. Class balancing was achieved by oversampling the minority class (random oversampling). The oversampling ratio and classification threshold were included in the hyperparameter search and optimized within the inner cross-validation (Table 3). No additional cost-sensitive loss function or class-weighting strategy was evaluated. The XGBoost tuned model used the ,,robustify“ pipeline for data processing, provided by the MLR3 package^29^. The graph for the tuned Ranger model included class balancing and scaling only. Hyperparameter optimization was performed using random search with default tuning spaces (Table 3). Parameters eta, lambda, and alpha were sampled on a logarithmic scale, whereas all other parameters were sampled on their original scale. Hyperparameter optimization in benchmark experiments necessitates nested resampling to mitigate bias in future performance prediction^30,31^. We employed a 10-fold cross-validation for outer resampling and a 5-fold cross-validation for inner resampling. All tuned models were trained on the area under AUC or BACC. Benchmark results were numerically reported using the AUC, BACC, sensitivity, specificity, logarithmic loss (Log Loss), and precision-recall AUC (PR-AUC). The performance of the best classifier in the benchmark experiment was visually presented using the receiver operation characteristic (ROC) curve and the confusion matrix. All analyses were conducted in R version 4.5.2 (2025-10-31) on a MacBook Pro equipped with 8 GB RAM. Model tuning used nested resampling with 5-fold inner and 10-fold outer cross-validation and a runtime terminator of 5,000 s for each tuning procedure. Actual total computation time was not systematically recorded. As a supplementary analysis, probability calibration was assessed using 10-fold cross-validated Platt scaling and isotonic regression, and calibration performance was quantified with the Brier score.

**Table 3:** Default search space for the tuned XGBoost classification model used for hyperparameter tuning with a random search algorithm.

XGBoost param	Lower	Upper	Levels
Oversample ratio	1	6	Inf
Nrounds	1	5,000	5,000
Eta	0.0001	1	Inf
Max depth	1	20	20
Colsample bytree	0.1	1	Inf
Colsample bylevel	0.1	1	Inf
Lambda	0.001	1,000	Inf
Alpha	0.001	1,000	Inf
Subsample	0.1	1	Inf
Threshold	0	1	Inf

Parameters were sampled on the original scale, with logarithmic scaling applied for eta, lambda, and alpha where appropriate^32^.

### Feature importance and external validation

Global feature importance, derived by averaging the absolute Shapley values across observations. Uncertainty was quantified using bootstrapped 95% confidence intervals. Estimates were obtained on the pooled external validation datasets (2021, 2022, and 2023)^33^. Feature stability was assessed via bootstrap resampling (B = 200). In each bootstrap replicate, the model was refit and SHAP values were computed for M = 30 randomly selected validation observations using Shapley sampling with sample.size S = 200. Features were then ranked by mean absolute SHAP importance. To assess the generalization ability of the final model, external validation was performed on the three validation datasets separately. Model predictions on these datasets were compared with ground-truth labels provided by the Olympic Testing Centre Brandenburg for 2025. Validation performance was quantified using AUC and BACC as primary metrics, and PR-AUC to better evaluate performance on the minority (positive) class. Confusion matrices were also reported to provide detailed insight into class-specific misclassification patterns. Covariate and prevalence shifts between the train/test (1992 to 2019) and external validation datasets (2021 to 2023) were examined by assessing changes in predictor distributions and outcome prevalence. Continuous predictors were standardized using training-set parameters, and multivariate distributional differences were quantified with maximum mean discrepancy (RBF kernel)^34^. Univariable covariate shift was assessed by comparing numeric predictors between the train/test and external validation datasets using Kolmogorov-Smirnov tests with bonferroni correction and Hedges’ g. In addition, cohort-specific positive outcome rates were calculated to evaluate prevalence shift.

## Results

**Fig. 2:**
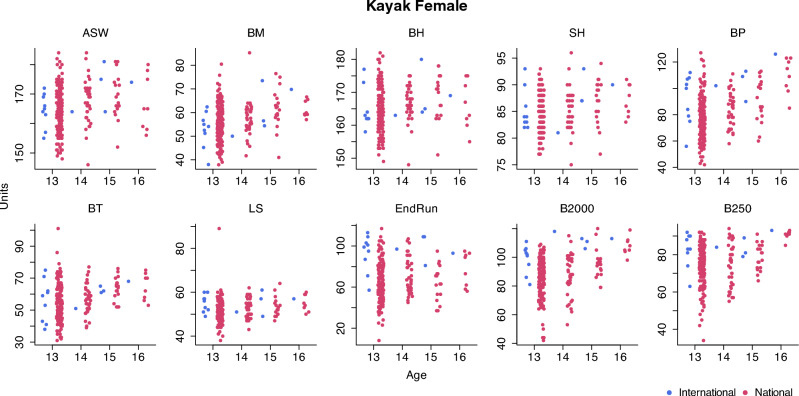
Anthropometric, physical fitness, and sport-specific performance variables across age in young female kayak athletes (train/test; n = 234), grouped by later national (red) and international (blue) senior performance level. Note: ASW = arm span width [cm], BM = body mass [kg], BH = body height [cm], SH = sitting height [cm], BP = bench press/pull, BT = ball throwing, LS = linear sprint, EndRun = endurance run, B2000 = 2000 m on-water canoe sprint time, B250 = 250 m on-water canoe sprint time. Anthropometric variables are shown in their original units, whereas physical fitness and sport-specific performance variables are shown as interval-scaled points.

**Fig. 3:**
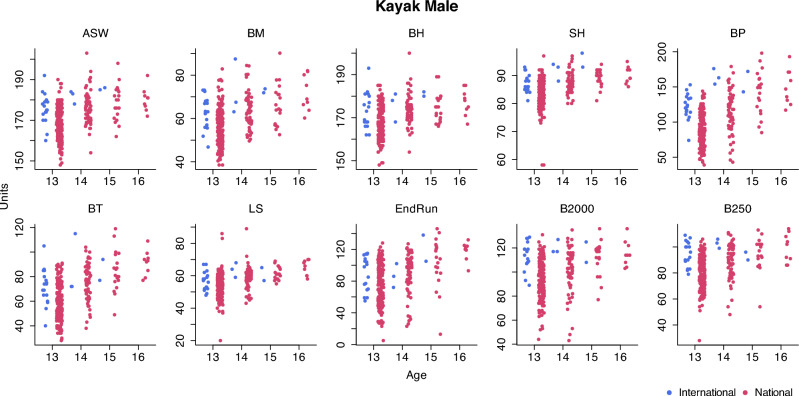
Anthropometric, physical fitness, and sport-specific performance variables across age in young male kayak athletes (train/test; n = 290), grouped by later national (red) and international (blue) senior performance level. Note: ASW = arm span width [cm], BM = body mass [kg], BH = body height [cm], SH = sitting height [cm], BP = bench press/pull, BT = ball throwing, LS = linear sprint, EndRun = endurance run, B2000 = 2000 m on-water canoe sprint time, B250 = 250 m on-water canoe sprint time. Anthropometric variables are shown in their original units, whereas physical fitness and sport-specific performance variables are shown as interval-scaled points.

**Fig. 4:**
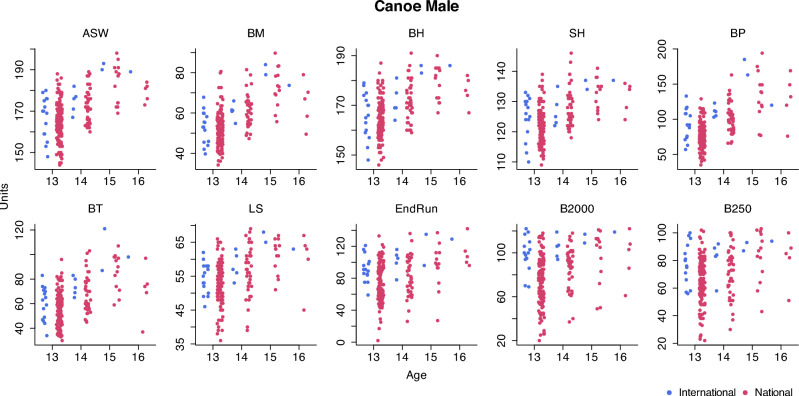
Anthropometric, physical fitness, and sport-specific performance variables across age in young male canoe athletes (train/test; n = 205), grouped by later national (red) and international (blue) senior performance level. Note: ASW = arm span width [cm], BM = body mass [kg], BH = body height [cm], SH = sitting height [cm], BP = bench press/pull, BT = ball throwing, LS = linear sprint, EndRun = endurance run, B2000 = 2000 m on-water canoe sprint time, B250 = 250 m on-water canoe sprint time. Anthropometric variables are shown in their original units, whereas physical fitness and sport-specific performance variables are shown as interval-scaled points.

Figures 2, 3 and 4 show the raw data of scores of the dataset (train/test) achieved in anthropometry, physical fitness, and sport-specific performance tests depending on peak SPL.

### Benchmark experiment

**Fig. 5:**
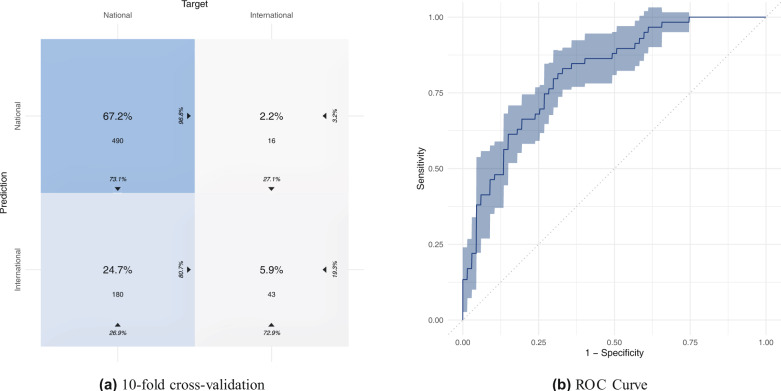
Performance of the tuned XGBoost classifier for predicting senior performance level in young canoe sprint athletes. Left: confusion matrix from 10-fold cross-validation (out-of-fold predictions: TP = 43, FN = 16, FP = 180, TN = 490). Right: ROC curve summarizing discrimination performance.

Table 4 presents the benchmark results of various classification models used to predict SPL based on a set of features comprising anthropometric, physical fitness, and sport-specific performance data. Among the evaluated models, XGBoost tuned on balanced accuracy showed the highest balanced accuracy (0.73) and the highest PR-AUC (0.35). However, the low PR-AUC indicates limited precision in identifying international athletes, even though sensitivity (0.73) and specificity (0.73) were balanced at the chosen threshold (0.07). The highest AUC was achieved by XGBoost tuned on AUC (0.83), while the lowest log loss came from untuned Ranger (0.24) but with zero sensitivity (Table 4). Figure 5 shows the confusion matrix and ROC curve for the XGBoost tuned on balanced accuracy classification model from the benchmark experiment. From the outer resampling, we selected the XGBoost (tuned on BACC) with the highest balanced accuracy and refit it on the full dataset (1992 to 2019). As a supplementary analysis, post hoc calibration slightly improved the Brier score from 0.077 to 0.070 using Platt scaling and to 0.068 using isotonic regression, but calibrated probabilities were not used for external prediction.

**Table 4:** Benchmark results of various classification models used to predict senior performance levels based on a feature set comprising anthropometric measurements, physical fitness data, and sport-specific performance metrics.

Model	AUC	BACC	Log loss	PRAUC	Sensitivity	Specificity	PPV	NPV
Rpart	0.69 [0.54, 0.82]	0.50 [0.47, 0.56]	0.30 [0.24, 0.35]	0.16 [0.10, 0.21]	0.03 [0.00, 0.17]	0.96 [0.94, 0.99]	0.05 [0.00, 0.24]	0.92 [0.91, 0.93]
Ranger	0.79 [0.69, 0.90]	0.50 [0.50, 0.50]	0.24 [0.20, 0.28]	0.27 [0.13, 0.44]	0.00 [0.00, 0.00]	1.00 [1.00, 1.00]	NaN [NA, NA]	0.92 [0.92, 0.93]
Featureless	0.50 [0.50, 0.50]	0.50 [0.50, 0.50]	0.28 [0.26, 0.28]	0.08 [0.07, 0.08]	0.00 [0.00, 0.00]	1.00 [1.00, 1.00]	NaN [NA, NA]	0.92 [0.92, 0.93]
Ranger tuned on auc	0.79 [0.70, 0.90]	0.57 [0.49, 0.71]	0.28 [0.23, 0.34]	0.30 [0.15, 0.40]	0.32 [0.00, 0.93]	0.83 [0.37, 1.00]	0.18 [0.01, 0.46]	0.94 [0.92, 0.99]
Ranger tuned on bacc	0.79 [0.69, 0.91]	0.73 [0.61, 0.88]	0.28 [0.22, 0.33]	0.32 [0.14, 0.52]	0.78 [0.52, 1.00]	0.68 [0.60, 0.79]	0.18 [0.13, 0.29]	0.97 [0.94, 1.00]
XGboost tuned on auc	0.83 [0.76, 0.90]	0.64 [0.50, 0.79]	0.26 [0.21, 0.33]	0.31 [0.18, 0.47]	0.40 [0.00, 0.96]	0.88 [0.50, 1.00]	0.36 [0.13, 0.90]	0.95 [0.92, 1.00]
XGboost tuned on bacc	0.81 [0.70, 0.90]	0.73 [0.64, 0.80]	0.26 [0.19, 0.35]	0.35 [0.15, 0.59]	0.73 [0.54, 0.83]	0.73 [0.62, 0.83]	0.20 [0.15, 0.26]	0.97 [0.95, 0.98]

*AUC* area under receiver operation curve, *BACC* balanced accuracy, *PR-AUC* precision-recall AUC, *PPV* positive predicted value, *NPV* negative predicted value. Wide intervals for sensitivity and PPV, particularly for minority-class predictions, reflect the small number of international athletes and should be interpreted with caution

### Feature importance

**Fig. 6:**
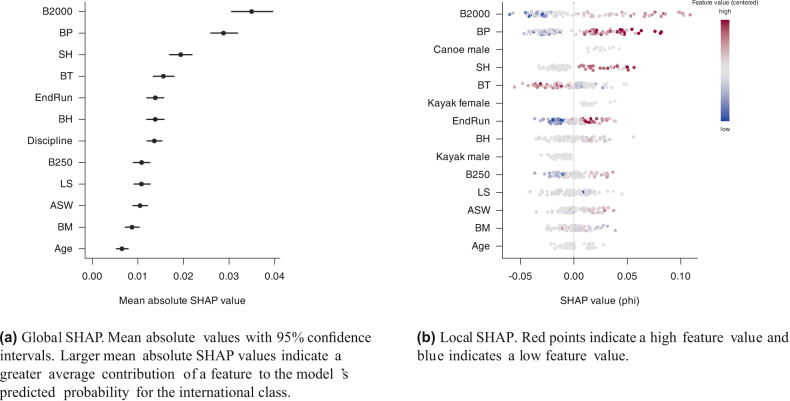
Global (left) and local (right) SHAP results for the final tuned XGBoost classifier predicting senior performance level in young canoe sprint athletes from anthropometric, physical fitness, and sport-specific performance variables. Note: ASW = arm span width, BM = body mass, BH = body height, SH = sitting height, BP = bench press/pull, BT = ball throwing, LS = linear sprint, EndRun = endurance run, B2,000 = 2,000 m on-water canoe sprint time, B250 = 250 m on-water canoe sprint time.

Feature importance on the validation dataset suggested a relatively broad set of relevant predictors (Fig. 6). Shapley analyses on the external validation dataset highlighted 2,000 m on-water canoe sprint time, 2 min bench press and bench pull test, and sitting height as the three most influential features, indicating that these variables are particularly important for the model’s out-of-sample predictions. The bootstrap ranking indicated high stability for the top predictor (B2,000; median rank 1, 95% percentile interval 1-2), whereas most other features showed wide rank intervals, suggesting limited stability of their relative importance (Table 5). Therefore, predictors with wide bootstrap rank intervals should not be over-interpreted as robust determinants of SPL.

**Table 5:** Bootstrap-based stability of global SHAP feature importance for the tuned XGBoost model.

Feature	Rank median	2.5% percentile	97.5% percentile
B2000	1	1	2
BP	2	1	6
EndRun	5	2	11
BH	6	3	12
Age	6	2	12
SH	6	2	11
B250	7	2	12
BT	7	3	12
Discipline	8	3	12
LS	9	3	12
ASW	10	4	12
BM	10	5	12

Features are ranked within each of 200 bootstrap refits by mean absolute SHAP value computed on 30 randomly selected validation observations (Shapley sample.size = 200). The table reports the median rank and the 2.5th to 97.5th percentile interval across bootstrap replicates. *ASW* arm span width, *BM* body mass, *BH* body height, *SH* sitting height, *BP* bench press/pull, *BT* ball throwing, *LS* linear sprint, EndRun = endurance run, B2,000 = 2,000 m on-water canoe sprint time, B250 = 250 m on-water canoe sprint time

### External validation

**Fig. 7:**
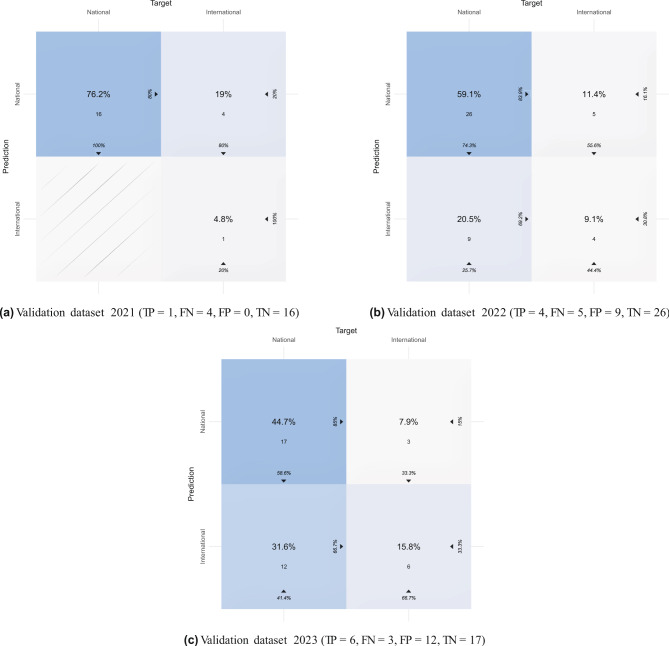
Confusion matrices for the external validation datasets (2021 to 2023). Cells show absolute counts of correctly and incorrectly classified international and national athletes for the tuned XGBoost model.

The XGBoost tuned on BACC model was evaluated on validation datasets from 2021 (n = 21), 2022 (n = 44), and 2023 (n = 38). AUCs ranged from 0.68 to 0.73, balanced accuracy from 0.60 to 0.63, and precision-recall AUC (PR-AUC) values were 0.46, 0.31 and 0.50 for 2021, 2022 and 2023, respectively (Table 6). Confusion matrices with absolute counts for each validation set are provided in the Fig. 7. Multivariat covariate shift between the training dataset and the external validation datasets was not statistically significant. The first-order maximum mean discrepancy values were 0.266 for 2021, 0.239 for 2022, and 0.473 for 2023, indicating the largest distributional deviation in the 2023 validation cohort. Univariable covariate shift between the development period (1992 to 2019) and the validation cohorts (2021 to 2023; mean Hedges’ |*g*| = ${0.45\pm 0.38}$) were observed. Nine of 33 comparisons remained significant after Bonferroni correction. The strongest shifts were observed for age (|*g*| = 1.01), ball throwing (|*g*| = 0.63), bench press/pull (|*g*| = 0.52), and 250 m on-water canoe sprint time (|*g*| = 0.50). The positive outcome prevalence was 8% in the training cohort and higher in the external validation cohorts, at 23.8% in 2021, 20.5% in 2022, and 23.7% in 2023.

**Table 6:** External validation of the XGBoost model on datasets from 2021 to 2023.

Validation set	AUC	BACC	Log loss	PRAUC	Sensitivity	Specificity	PPV	NPV
2021	0.68	0.60	0.82	0.46	0.20	1.00	1.00	0.80
2022	0.69	0.59	0.59	0.31	0.44	0.74	0.31	0.84
2023	0.73	0.63	0.57	0.50	0.67	0.59	0.33	0.85

*AUC* area under receiver operation curve, *BACC* balanced accuracy, *PR-AUC* precision-recall AUC, *PPV* positive predicted value, *NPV* negative predicted value.

## Discussion

The aim of this study was to evaluate whether machine learning models comprising anthropometric, physical fitness, and sport-specific performance data from young canoe sprint athletes can predict their senior performance level (SPL). Our main findings indicated that (i) an XGBoost model tuned on balanced accuracy achieved acceptable discrimination, balanced accuracy, and low precision for predicting SPL in young canoe sprint athletes (ii) the most important features for SPL prediction were sport-specific performance (i.e. 2,000 m on-water canoe sprints) and physical fitness tests (i.e. 2 min bench press and bench pull test), and (iii) the XGBoost model tuned on balanced accuracy showed limited temporal generalizability, with moderate discrimination, low precision, and modest but consistently above-chance balanced accuracy across three validation cohorts (2021, 2022, and 2023). The first main finding was that the tuned XGBoost model showed good overall discrimination and balanced accuracy for predicting SPL in junior canoe sprint athletes based on anthropometric, physical fitness, and sport-specific performance data (AUC = 0.81; BACC = 0.73; Table 4). However, precision for the international class was low (PRAUC = 0.35, PPV = 0.20), indicating many false-positive international predictions. This pattern is plausible, as the model likely identifies a broader pool of promising athletes, of whom only a small proportion ultimately attain international funding status. This result is partly in line with findings from other studies in other sporting disciplines. Studies in other sports (e.g. track and field, alpine skiing, basketball, handball) have reported that junior performance does not necessarily predict SPL^3,5,21^. For example, a systematic review and meta-analysis of retrospective and prospective studies found that only a small proportion of senior athletes (2 to 60%) had previously achieved an equivalent performance level as juniors, and only a small proportion of junior athletes ( 6 to 41%) later reached an equivalent level at senior age, with these proportions decreasing as junior age category decreased and performance level increased (i.e. national [tier 3], international [tier 4], world-class [tier 5])^19^. The international prevalence in our validation cohorts ( 20 to 24%) lies within the range of 6 to 41%, suggesting that the observed class imbalance reflects realistic talent-development proportions. Therefore, low precision should be interpreted in this context rather than solely as model failure. However, these estimates are only partly comparable to our sample, which reflects the transition from junior national-level development to later senior performance status in German canoe sprint. Another recent study used a machine learning approach including physical and psychological variables to predict talent selection (i.e. staying at the academy for the forthcoming season) in young male academy soccer players with moderate predictive performance^24^. However, a recent review concluded that machine learning methods for talent identification in team sports may complement, but not replace, traditional approaches, while reported discrimination ranged from modest to excellent and many studies showed a high risk of bias due to inadequate analyses and limited generalisability^35^. In individual CGS-type sports, machine learning may predict SPL more accurately because performance depends more on measurable physiological and sport-specific capacities and on objective outcomes such as distance and time. For example, in swimming, young athletes with higher performance levels were more likely to achieve higher performances during the later course of LTAD^36,37^. More specifically, it was reported in a retrospective analysis that the groups who were ultimately less successful had reached only 70% of the performance of the successful groups at the age of 12 to 13 years and only 57% during their subsequent athletic development^36^. Moreover, it has been shown that the best junior swimming times at international level (junior world championships) explained 59% of the variance in identifying the top 30% performers at senior world championships^37^. In canoe sprint, previous work in national-level athletes has shown that adding anthropometric and physical fitness variables to demographic data improves the prediction of race time, which supports the relevance of physiological and sport-specific capacities for performance^6^. However, as TI and TLTAD are multidimensional processes, additional information on psychological, sociodemographic, and maturity-related factors may further improve prediction quality. Discrepancies between study findings may be explained by differences in sport discipline, data sources and methodological approaches. Additionally, in our study, class imbalance (few international athletes) likely contributed to the low precision/high false-positive rate and may limit generalizability if the prevalence of international athletes differs across cohorts, thereby affecting precision. In addition, model performance may depend on the underlying class distribution and the selected classification threshold, both of which influence the trade-off between false-positive and false-negative decisions in practical talent selection. This was also reflected in our benchmark, where the models were generally better at ruling out future international athletes than at correctly identifying them. Also, it is important to note that “talent is not a stable characteristic”^38^, and a predicted probability >50% of achieving international SPL in the future does not guarantee that an athlete following a talent development pathway will become an international-level athlete. In summary, the XGBoost model is better in ruling out future international athletes than correctly identifying them and therefore limits the practical utility.

Regarding our second main finding, the most important feature predicting SPL in the model was a sport-specific performance test (i.e. 2,000 m on-water canoe sprints). Muscular endurance (i.e. 2 min bench press and bench pull test) also ranked highly on average, but with lower stability across bootstrap samples (Table 5). This is partly consistent with results from a previous study, which found that bench press and pull tests were highly associated with the race time around test time period in national-level (tier 2) junior canoe sprint athletes^6^. The importance of 2,000 m performance may be explained by the fact that this distance reflects sport-specific endurance. However, sport-specific tests may already reflect the talent and performance capacity that later define senior success, introducing potential circularity. Therefore, 2,000 m performance should be interpreted as a marker of current sport-specific ability rather than an independent causal predictor. This also raises the question of whether a reduced model excluding on-water performance would retain sufficient practical utility for early talent identification, particularly in younger age groups in whom sport-specific performance may be less fully developed. This should be addressed in future studies. Overall, the results suggest that both sport-specific endurance and muscular endurance are important for SPL classification. Previous studies in highly-trained and national-level canoe sprint athletes also included anthropometric variables in the models to predict canoe race time^6,14,15^. While other anthropometric measure (e.g. arm span width, body mass) appear to play only a minor role in our classification model (as indicated by the feature importance analysis), the information of these parameters may still be relevant in other modeling settings. In support of this notion, skeletal muscle mass was a significant predictor in linear regression models using a feature set of demographics, anthropometrics, and physical fitness data to predict 500 m and 2,000 m canoe race time in national-level (tier 2) junior athletes^6^. The different importance of the features could be explained by the distribution of national and international SPL labels among the variables of our dataset (Figs. 2, 3, and 4). Visual inspection indicated that anthropometric data of athletes who reached international SPL were equally distributed and not predominantly located at the upper or lower end of the distribution. Although feature importance was primarily derived from SHAP analyses on a single validation dataset, 2000 m performance was consistently supported, also supported by the built-in XGBoost importance and Breiman’s permutation feature importance. Nevertheless, the reported rankings should be interpreted as model-specific and non-causal. Based on these results it is important to note that these findings do not imply to only exercise these variables (e.g. 2,000 m on-water canoe sprints and 2 min bench press and bench pull test) during LTAD anymore. Common approaches should be used to lay a foundation of physical fitness for subsequent sport-specific performance^1^. To sum up, the most important feature identified by the Shapley values on the validation datasets was 2,000 m on-water canoe sprints.

The third main finding was that the XGBoost model tuned on balanced accuracy showed limited temporal generalizability, with moderate discrimination (AUC: 0.68 to 0.73), modest but consistently above-chance balanced accuracy (0.59 to 0.63), and moderate but variable sensitivity (0.20 to 0.67) across three external validation datasets (2021 to 2023). However, precision (PPV) for identifying international athletes was low overall (2022: 0.31; 2023: 0.33), indicating a high false-positive rate, whereas in 2021 precision was 1.00 but based on only one positive prediction. This means that if the model flags 10 athletes as future internationals, only about 3 will actually reach that level (PPV: 0.30), while additional future internationals will remain undetected because the model identifies only about 40% of all athletes who later achieve international status (sensitivity: 0.40). Practically, this implies that most “international” recommendations would be incorrect, increasing downstream assessment burden and resource use in talent pathways. A possible explanation is that the international outcome captures only a selected subset of athletes with high underlying potential. Some athletes with similar potential may not reach this level because of selection decisions, injuries, dropout, or other contextual factors, reducing class separability and making the international class harder to identify. This trade-off was also influenced by the classification threshold selected during the tuning process. Another possible explanation is that relevant predictors were not available in our dataset, particularly measures of psychological characteristics, biological maturation status, and sociodemographic. Sensitivity may also vary across years because older athletes are closer to ultimate career achievement, which can increase separation in performance-related predictors and make correct classification easier. It is also possible that cohort strength differed between years (i.e. some weaker cohorts), which could change class separability and thereby affect sensitivity in external validation. Multivariate covariate shift between the train/test and external validation datasets was not statistically significant. However, first order maximum mean discrepancy was highest in 2023 indicating somewhat greater distributional differences compared to 2021 and 2022 with train/test dataset. These shifts may have increased predicted probabilities for the international class and may partly explain the higher sensitivity but lower specificity observed in 2023. Observed changes in international prevalence (train/test: 0.08; 2021: 0.23; 2022: 0.20; and 2023: 0.24) may also partly explain the limited temporal stability and year-to-year variability and precision. The higher log loss in 2021 may reflect the smaller validation sample (n = 21) and younger age distribution (${13.7\pm 0.56}$ years) compared with 2022 (${14.0\pm 0.88}$ years) and 2023 (${15.03\pm 0.85}$ years) leading to less reliable probability estimates. In 2021, the model showed a conservative prediction pattern, identifying only one international athlete while correctly classifying all national athletes. This may partly reflect the younger age distribution and lower values in several performance-related variables, which likely reduced predicted probabilities for the international class. Pandemic-related disruption may also have affected training, competition exposure, and athlete development, particularly in the 2021 cohort, but could not be verified. Although the model performed better than chance overall, the low sensitivity in 2021 indicates that statistical performance does not necessarily translate into sufficient practical utility for talent identification. Ongoing validation is required to ensure stability over time and support valid usability in practice. Due to the low precision for the international class across the external validation datasets, the model should be interpreted as an supportive screening tool rather than a standalone selection instrument.

The present study comes with some limitations that need to be acknowledged and discussed. For instance, although several demographic, anthropometric, and fitness data were included, we were not able to include information on maturity status, psychological characteristics, and sociodemographic characteristics. It is well-known that chronological age is not a good indicator of development and maturation with consequences for training programming^39^. Because skeletal age or estimated PHV were unavailable, biological maturation could not be modeled separately from chronological age. Nevertheless, chronological age was a significant predictor in the regression model for estimating race times in contrast to maturity status (i.e. years from peak-height velocity)^6^. Relatedly, sitting height may have partially acted as a crude proxy for maturation-related body proportions. However, our bootstrap SHAP stability analysis indicated that its importance was not robust (median rank 6; 95% rank interval 2-11), underscoring that anthropometric proxies cannot replace established maturity indicators (e.g.skeletal age, estimated PHV). Future prediction models may benefit from including established maturity indicators, such as skeletal age or estimated peak-height velocity, and from modelling longitudinal performance trajectories. Additionally, feature importance was estimated using Shapley values on a single validation dataset. Given that SHAPLEY-based importance can be sensitive to model specification, correlated predictors, and sampling variability, the stability and magnitude of individual predictors may be overestimated, so the reported importance rankings should be interpreted with caution and validated in independent samples. In addition, the long observation period may have introduced cohort effects related to changes in training systems, selection procedures, or competition structures over time. Also, athletes had different follow-up periods and some younger athletes in the validation sets may not yet have reached their peak career level by 2025. Another limitation is that some athletes in the validation datasets contributed repeated observations, without mixed-effects or grouping adjustment, which may have reduced statistical independence and affected validation metrics. Because data on non-nominated athletes were unavailable, potential coach-based selection bias cannot be quantified, limiting generalisability to all young canoeists. Furthermore, the sample was drawn from a single country and external validation was limited to athletes from the same regional system, which restricts generalisability to other regions or countries. Moreover, the definition of international SPL was based on the German national cadre system and may therefore be partly country-specific, which could further limit transferability to other sport systems with different structures and selection criteria. In this context, the proprietary interval-scaled scoring system may also limit generalizability to settings using raw measurement units or different normative systems. In addition, detailed weather conditions during the outdoor tests were not consistently available, and environmental variation may therefore have influenced the performance. Finally, individual load selection in the bench press and bench pull tests may partially have been influenced by lack of objectivity. Individual load distributions could not be reconstructed retrospectively, which may limit comparability of the resulting strength indices across athletes. However, the measurements were always conducted by experienced assessors. Although the dataset was collected within a structured testing system, full standardization of measurements across years could not be verified retrospectively, which may have affected data comparability over time^40^.

## Conclusions

In the present study, we have developed a classification model to predict SPL of young canoe sprint athletes based on a set of features comprising anthropometric, physical fitness, and sport-specific performance data. The used model predicted SPL with modest but consistently above-chance balanced accuracy, low precision, limited temporal generalizability, and variable sensitivity across validation years. The XGBoost model should not be used to select or deselect individual athletes for talent programmes. However, it may serve as a supportive screening tool to identify a broad pool of athletes who may benefit from additional monitoring or support, with the understanding that most athletes predicted to reach international level will be false positives. While these findings are particularly relevant to LTAD in Olympic canoe sprinting, establishing a solid foundation of physical fitness during the early stages of LTAD is also essential for optimizing subsequent sport-specific performance. The present findings may help practitioners involved in talent selection and development in Olympic canoe sprinting and may inform the development of future prediction models for young canoeists based on anthropometric, physical fitness, and sport-specific performance data.

## Data Availability

The data are available upon request and with the permission of the Federal Canoeing Federation of Brandenburg.

## Declarations

### Accession codes

The code needed to reproduce the full analysis using the dataset used here can be found on GitHub: https://github.com/christian43/Canoe_Predictions.git.
